# EpiVECS: exploring spatiotemporal epidemiological data using cluster embedding and interactive visualization

**DOI:** 10.1038/s41598-023-48484-9

**Published:** 2023-12-01

**Authors:** Lee Mason, Blànaid Hicks, Jonas S. Almeida

**Affiliations:** 1 National Institutes of Health, Bethesda, USA; 2 Queen’s University Belfast, Belfast, UK

**Keywords:** Software, Machine learning, Data processing

## Abstract

The analysis of data over space and time is a core part of descriptive epidemiology, but the complexity of spatiotemporal data makes this challenging. There is a need for methods that simplify the exploration of such data for tasks such as surveillance and hypothesis generation. In this paper, we use combined clustering and dimensionality reduction methods (hereafter referred to as ‘cluster embedding’ methods) to spatially visualize patterns in epidemiological time-series data. We compare several cluster embedding techniques to see which performs best along a variety of internal cluster validation metrics. We find that methods based on k-means clustering generally perform better than self-organizing maps on real world epidemiological data, with some minor exceptions. We also introduce EpiVECS, a tool which allows the user to perform cluster embedding and explore the results using interactive visualization. EpiVECS is available as a privacy preserving, in-browser open source web application at https://episphere.github.io/epivecs.

## Introduction

A key part of public health research is analyzing how diseases are distributed across space^1,2^. This can help epidemiologists allocate resources, discover potential risk factors, measure health disparities, implement targeted public health policy, and more^1,3,4^. Typically, such analysis considers a specific point or period in time and does not explicitly model or analyze temporal structures in the data, despite the fact that many datasets in epidemiology are both spatially and temporally referenced. Analyzing time alongside space can provide useful insights that spatial analysis alone cannot^5–7^, including an improved understanding of communicable disease transmission, a more comprehensive representation of differences in epidemic response, and a way to compare the impact of varying public health interventions^1,8–11^. Despite recent advances and increased interest in spatiotemporal analysis, the complexity and scale of the data can make it difficult for analysts to perform such analysis effectively^5,6,12^.

The complexity of spatiotemporal analysis is especially challenging for exploratory tasks, such as disease surveillance and hypothesis generation, because it makes it difficult to rapidly investigate insights at scale^11^. For this reason, it is useful to have methods that allow analysts to find and explore patterns in spatiotemporal data with speed and flexibility. One particularly effective way of exploring complex data is by using interactive visualizations^13^. A well-designed visualization takes advantage of the analyst’s visual processing skills to convey complex and nuanced information that would not be apparent in an alternative format (e.g. tabular)^13^. The addition of interaction means that the analyst can display the details which are most important to them, and follow-up on any potential insights they gain from the resulting visualizations. Interaction is especially important for complex datasets because it means a lot of information can be included without needing to visualize it all at once^14,15^. Spatiotemporal data, in particular, is challenging to visualize because both spatial and temporal data are often best represented using the ‘position’ visual channel, and it is therefore difficult to visualize both together^16^. Interaction addresses this challenge by limiting how much spatial or temporal data is visualized at once, in a manner that is guided by user engagement^16^. However, hiding too much data behind interaction can impede one of the main benefits of multivariate exploratory analysis: uncovering interesting patterns that span the dataset. This motivates the use of methods capable of simplifying the data in such a manner that it can be visualized globally, providing some high-level insights before the analyst explores more granular patterns using interaction.

Unsupervised learning techniques can be used to gain a high-level summary of spatiotemporal data by uncovering potentially informative patterns^12^. One popular technique is clustering—finding subsets of the data which show some degree of internal similarity. Clusters act as a summary of the dataset, simplifying the initial information that the analyst must consider. In an interactive dashboard, additional details can then be uncovered through interaction. In epidemiology, time-series clustering has been used for tasks such as surveilling infectious diseases, estimating transmission dynamics, and disease forecasting^17–19^. There are many different clustering algorithms available, each having subtle strengths and weaknesses, which makes it difficult to choose the best method for a given problem^20^.

Clustering methods provide an especially useful summarization of data when their output includes an informative representation of each cluster, e.g. the centroids in k-means^21^. Self-organizing maps, a basic machine-learning based clustering method, are particularly useful for data summarization because they provide both a high-dimensional and low-dimensional representation of the clusters^22,23^. For this reason, self-organizing maps are especially useful for visualization^24,25^. However, self-organizing maps require the low-dimensional representation of the clusters to be positioned on a fixed grid, which can impede both the clustering quality and the informativeness of the 2D cluster representation^26^. An alternative approach is to use any clustering technique which produces a vector representation of the clusters (e.g. the centroids in k-means) and then to calculate a 2D representation of those vectors using a dimensionality-reduction technique. This allows greater flexibility in the choice of both the clustering method and the dimensionality-reduction method, which if appropriately chosen can lead to better results. This is the approach taken by the oKMC+ method: the application of Sammon mapping (a non-linear dimensionality reduction technique) to k-means centroids, resulting in higher quality clusters and a better low-dimensional cluster representation when compared with self-organizing maps^26^. There is no standard terminology for methods that cluster data and produce a low-dimensional representation of the clusters, so we will refer to them here as ‘cluster embedding’ methods. In this paper, we will compare the performance of several cluster embedding methods on real-world epidemiological data. We will argue here that cluster embedding is a particularly effective route to explore both overall analytical context and fine-grained spatiotemporal patterns. We will also introduce a web-tool, EpiVECS, which implements an interactive approach to cluster embedding.

## Results

### Method comparison

#### Data

The primary goal of this work is to simplify the analysis of real-world spatiotemporal epidemiological data, and therefore we have assessed the proposed methodology using publicly available datasets from the US Centers for Disease Control and Prevention (CDC). The following empirical results are best understood by experimenting with the accompanying EpiVECS web application in conjunction with the supplementary Observable Notebooks. In both cases, the proposed methodology can be tested interactively without the need to download or install any software. To ensure that the methods are tested on a variety of scenarios, we have included 14 datasets covering different concepts including cancer mortality rates, COVID-19 patient bed utilization, and age-adjusted all-cause mortality rates. The datasets include US county-level and state-level spatial resolutions, and week-level, month-level, and year-level temporal resolutions. A range of vector lengths are present, the shortest being of length 17 and the longest of length 171. The datasets were filtered to ensure that there are no missing values; specifically, for each dataset, a time-range was chosen and areas with missing values in that time-range were excluded. For information on the datasets and how they were collected and processed, see Availability.

#### Clustering validation

To compare the performance of the cluster embedding methods, we first compare the performance of the clustering methods (self-organizing maps and k-means) using several internal cluster validation metrics. Internal cluster validation metrics attempt to quantify cluster quality using internal properties of the clusters such as cluster separation. Many different internal validation metrics have been proposed and, like other parameters in multivariate exploratory analysis, different metrics perform best in different scenarios^27,28^. Therefore, it is helpful to use a variety of metrics to generate a comprehensive comparison between methods. In this work, we have chosen the following internal cluster metrics based on recommendations from comparison studies: Davies-Bouldin, Calinski-Harabasz, SDbw, and Silhouette Score, and mean square quantization error^27,28^.

**Figure 1:**
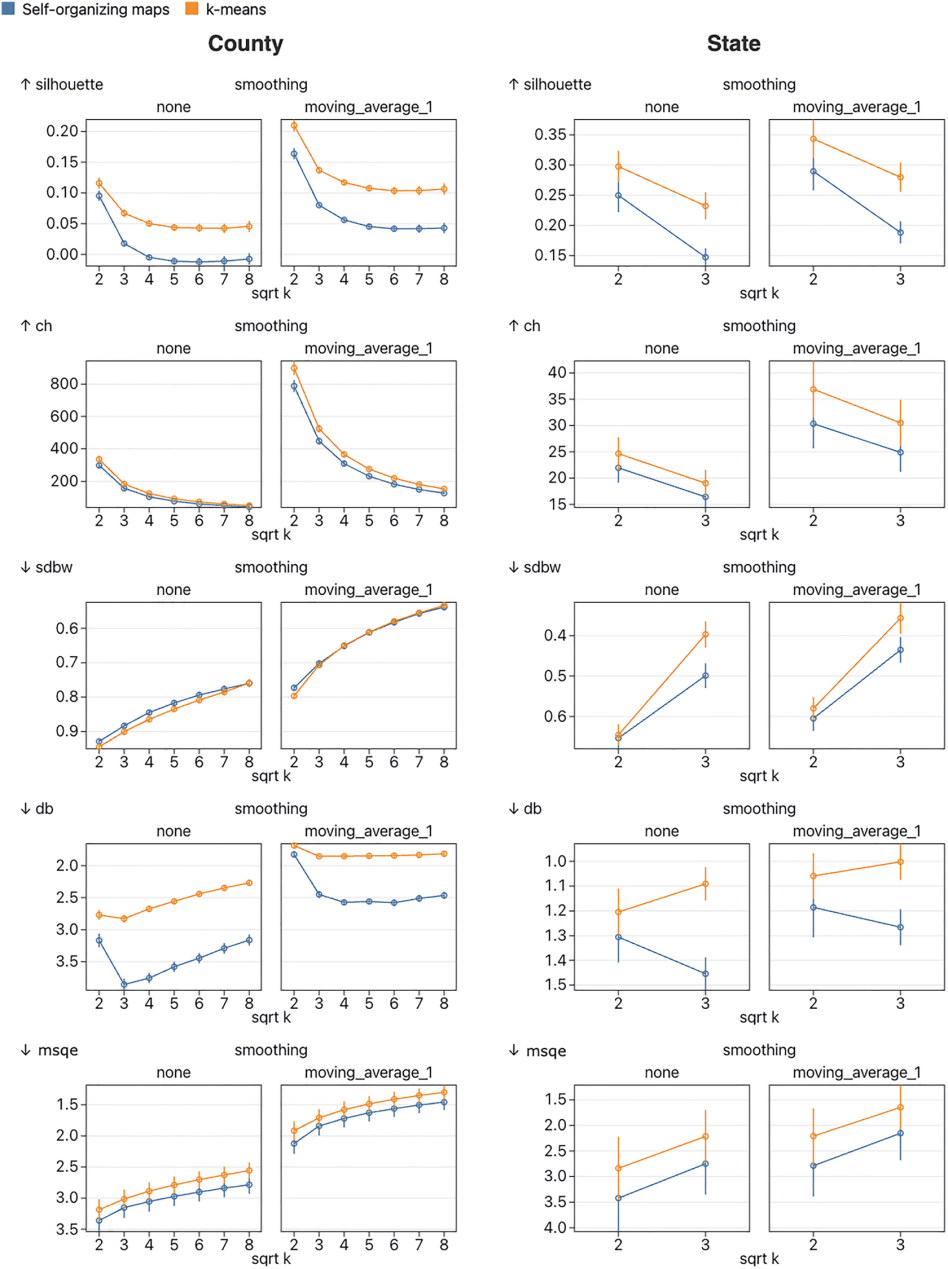
A comparison of the quality of the clusters produced by self-organizing maps and k-means clustering. Five internal cluster validation metrics are used: Silhouette, Calinski-Harabasz (CH), SDbw, Davies-Bouldin (DB), and mean square quantization error (MSQE). Each plot shows the mean values over all datasets in the relevant group (split by spatial resolution) and 8 repeat runs, with 95% confidence intervals. Each plot shows results for unsmoothed data and data smoothed using a simple moving average method with window width 3. Metrics which indicate better performance at lower values (SDbw, DB, and MSQE) are plotted on a reversed y-axis so that more favorable values are always plotted further up the y-axis. According to all metrics except SDbw, k-means scored better than self-organizing maps at all values of k. Using a two-sided t-test, most of these results are significant at the 0.05 significance level with a couple of notable exceptions, including MSQE which shows significant results only for unsmoothed county-level results at k of 49 and 64. Another notable exception is that SDbw doesn’t show a significant difference between methods for smoothed results with k greater than 4. Scores for smoothed data were better than unsmoothed data in all cases.

An important parameter in both k-means and self-organizing maps is the number of clusters. In k-means, the number of clusters is set by a single parameter (k), whereas in self-organizing maps it is determined by the size and shape of the grid. In this work, we limit self-organizing maps to a square grid and therefore all tested values of k will be square numbers. There is no definitive way to find an appropriate value for k and several popular methods rely on a subjective interpretation of a visualization (e.g. a Silhouette plot^29^). In the EpiVECS tool we leave the choice of k up to the user, and so it is important that we test several values of k when comparing methods (Fig. 1). We have elected to test the following k values: 4, 16, 25, 36, 49, and 64. In cases where the test dataset is small (e.g. the state level datasets consist of around 50 vectors) we test only 4 and 9. We keep other parameters for k-means and self-organizing maps fixed at the default values used in the chosen implementations, shown in the supplementary observable notebooks (see Availability).

#### Dimensionality reduction validation

**Figure 2:**
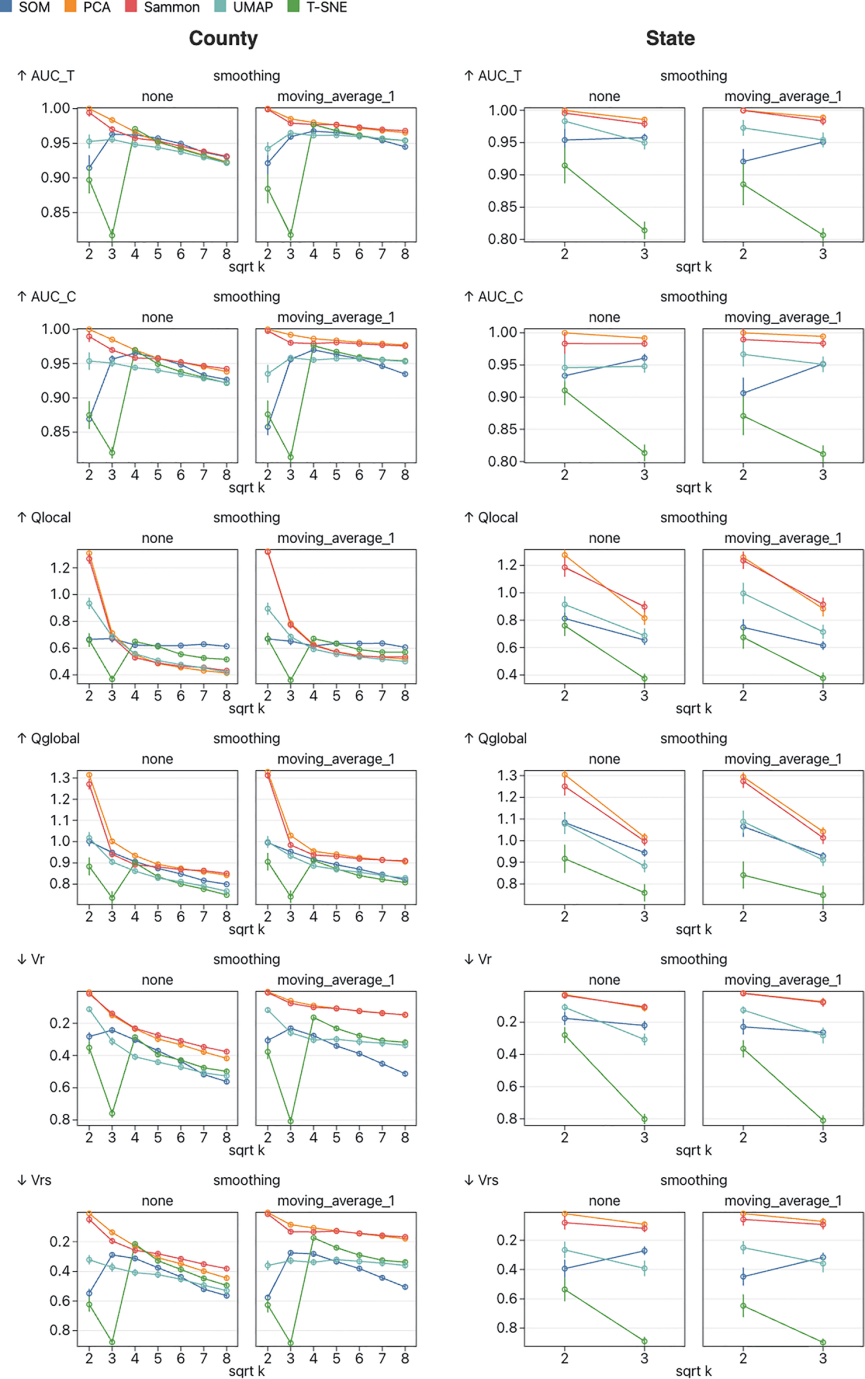
An evaluation of how well the 2D cluster representation matches the high-dimensional cluster representation, for each method. For self-organizing maps, the high-dimensional representation is the node weights and the 2D representation is the fixed location grid. For embedded k-means, high-dimensional representation is the cluster centroids and the 2D representation is these centroids embedded in 2D space using a variety of dimensionality reduction methods (tested here are PCA, Sammon mapping, t-SNE, and UMAP). The methods are tested using a variety of dimensionality-reduction validation metrics: AUC of trustworthiness (tAUC), AUC of continuity (cAUC), residual variance between the distance matrices using Pearson and Spearman correlation (VR and VRs respectively), co-k-nearest neighbor size (Qglobal), and local continuity meta criterion (Qlocal). Metrics which indicate better performance at lower values (VR and VRs) are plotted on a reversed y-axis so that higher values are always plotted further up the y-axis. Results are mixed and the relative performance of the methods depends on both the number of clusters k and the metric chosen. Sammon mapping and PCA perform similarly well and, with the exception of higher values of k according to the Qglobal metric, perform consistently better than the other methods. Surprisingly, the more modern UMAP and t-SNE performed comparatively poorly, but performance was better at higher values of k.

An important part of cluster embedding methods is producing an informative representation of the clusters in a low-dimensional (usually 2D) space. Ideally, the low-dimensional cluster representation should provide the analyst with some idea of how clusters relate to one another. One way this quality can be assessed is by using dimensionality-reduction validation metrics to quantify how well certain properties of the high-dimensional cluster representation are preserved in the low-dimensional cluster representation. There are many different validation metrics, and each assesses a different aspect of the dimensionality reduction, so it is useful to use a variety of metrics when comparing methods. In this work, we use AUC of trustworthiness (tAUC), AUC of continuity (cAUC), residual variance between the distance matrices (VR and VRs), co-k-nearest neighbor size (Qglobal), and local continuity meta criterion (Qlocal), all of which are implemented in the pyDRMetrics Python library^30^. A comparison of the dimensionality reduction quality of the implemented methods can be found in Fig. 2.

#### EpiVECS web-tool

EpiVECS is an open-source, in-browser web-tool which allows the user to perform cluster embedding on their own geospatial vector data and visualize the results in an interactive dashboard. All methods described in this paper are available as interactive features in this tool, alongside basic normalization, smoothing, and interpolation options. The user can choose from several default datasets, equivalent to those we have used here to compare the cluster embedding methods. The user can also provide and configure their own dataset using the data configuration wizard, which is designed to streamline the process of converting row data into vector data and linking it to geospatial information. The tool supports any numeric data so long as the Euclidean distance between the resulting vectors can be meaningfully interpreted by the analyst (other distance metrics are currently not supported). The user can also provide their own geospatial data in the GeoJSON format. Because the tool runs entirely in the browser, the data does not leave the user’s machine, which is useful for privacy preservation. However, at present the web libraries for performing many of the methods required by EpiVECS (e.g. clustering, dimensionality reduction) are not as mature as their counterparts in other languages such as Python and R, meaning they are not as efficient computationally or in terms of memory usage. This could present problems on very large datasets (> 10 million vectors), but from initial experimentation we suspect EpiVECS will be sufficient for most geospatial use-cases (see Supplementary Table S1 online).

**Figure 3:**
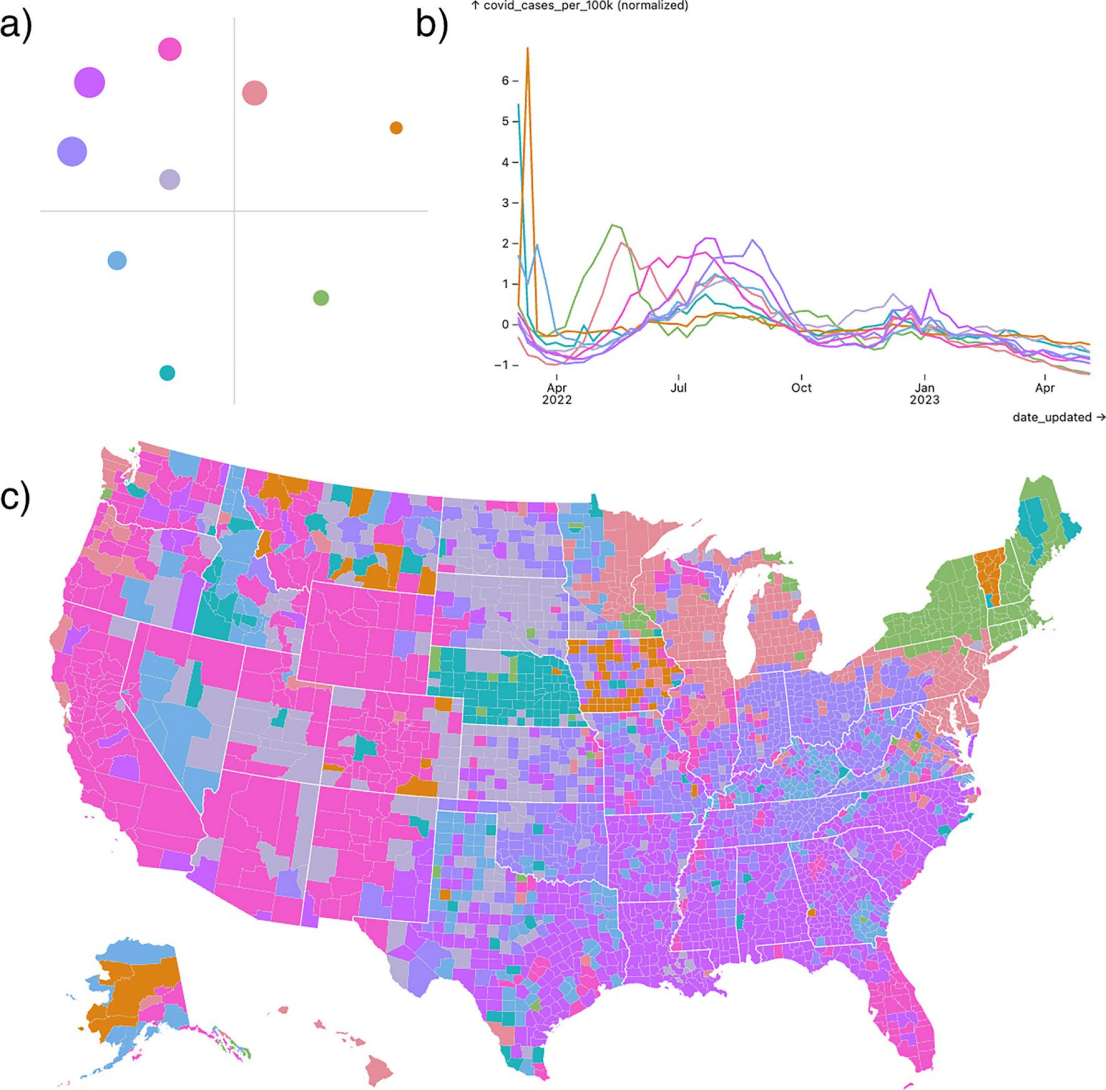
A screenshot of the EpiVECS tool with weekly county-level COVID-19 case rates. Each time-series has been z-score normalized space-wise. The cluster embedding method used was K-means + Sammon (also known as oKMC+) with 16 clusters. (**a**) The bubble plot shows the low-dimensional cluster representation, with the size of the bubble proportional to the number of vectors assigned to the corresponding cluster. Here, each bubble is the embedded k-means cluster centroid. (**b**) The line plot shows the high-dimensional cluster representation. Here, each line is the k-means cluster centroid. (**c**) A choropleth plot where each location is colored according to its assigned cluster. Note the clear spatial structure that emerges, e.g. the counties in the northeast US were generally assigned to the same cluster, except some counties in Vermont, which by looking at the line plot we can see correspond to counties with an unusually early first COVID peak. Close inspection reveals spatial correlation patterns that follow state and/or regional lines—as is the case for the state of Vermont and, differently, for the six states of the New England region.

The dashboard consists of three primary plots: a line plot showing the cluster centroids, a bubble plot showing the embedded cluster positions, and a choropleth (see Fig. 3). The plots in the dashboard are designed with ‘link and brush’ functionality, wherein the user can highlight and select elements in one plot and the interaction is reflected in all other plots. For example, if the user hovers over a cluster centroid, then all areas in the choropleth which were assigned that cluster centroid’s label are highlighted, and the position of the embedded cluster in the bubble plot is also highlighted. The user can click on elements to select multiple labels which are kept highlighted; this selection can be cleared by clicking in an empty space on any of the plots. If the user hovers over any area in the choropleth, that area’s specific (normalized) vector is shown in the line plot—this shows how well areas match their assigned cluster centroids. Tooltips provide additional information tailored to the plot. We encourage the reader to experiment with the tool to see this functionality in action.

## Discussion

In this manuscript, we have investigated the use of cluster embedding to simplify the exploration of spatiotemporal epidemiological data. We compared two key methods: self-organizing maps and embedded k-means. For embedded k-means we compared four different dimensionality-reduction techniques to embed the cluster centroids: PCA, Sammon mapping, t-SNE, and UMAP.

Epidemiological time-series data is often characterized by properties which render analysis challenging, such as noise and non-linearity, and therefore we performed our experiments using real-world epidemiological data. A variety of clustering and validation metrics were used to ensure that the results were not sensitive to the choice of metric. Our results show that k-means clustering generally performs better than self-organizing maps, especially in regard to clustering quality. This confirms previous results by Flexer which showed that oKMC+ , an embedded k-means method with Sammon mapping, outperforms self-organizing maps in both the quality of clustering quality and dimensionality reduction quality^26^. Flexer took a different approach to assessment than the one described in this manuscript, using external validation metrics on simulated data rather than internal validation metrics on real-world data. This suggests that the superior performance of embedded k-means is consistent across a range of experimental designs. However, an important element in data exploration is the subjective experience and goals of the analyst, which these validation metrics are unable to assess. While self-organizing maps may result in lower quality clusters, the simplicity and convenience of the fixed grid structure may be desirable to the analyst. In the accompanying EpiVECS tool, we have included all methods discussed in this paper so that the analyst can experiment with different approaches.

There are two main issues to consider when using cluster embedding methods for data exploration: the methods will produce a result regardless of how meaningful it is, and the methods are sensitive to the choice of hyperparameters (especially the number of clusters k). These two issues are related because the choice of hyperparameters can result in a wide variety of different results. It is therefore difficult to assess the comparative merits of each configuration from numerical validation metrics alone. In contrast, presenting the results as an interactive visualization provides a mechanism to mitigate these issues because the analyst can explore the results in further detail, using analytical and domain knowledge to assess the results. To encourage this type of assessment, we have included a specific feature in the EpiVECS tool: when the user hovers over a specific area that area’s input time-series is shown alongside the centroid (or weight) of the cluster to which it was assigned. This allows the analyst to see how well different time-series match their assigned clusters to find out which set of clustering parameters recognize known spaciotemporal correlations.

Applying cluster embedding methods to time-series data comes with a number of challenges. The approach allows an analyst to explore temporal patterns, but it does not alone convey any information about the distribution of these temporal patterns over space. To provide that information, the accompanying EpiVECS tool includes a choropleth plot where each area is colored according to the corresponding time-series' assigned cluster. This approach requires a method of assigning colors to clusters, for example, by treating clusters as independent parameters and using a categorical color scheme. However, while this would convey the similarities between counties in each cluster, it would not convey how counties in different clusters relate to each-other. To address this, we used a key strength of cluster embedding: the summary of the similarities between clusters using positions in 2D space. This can be used to generate a more meaningful assignment of colors reflecting both relationships within and between clusters. Specifically, the EpiVECS tool maps the 2D point representation of the clusters onto a cylindrical color space. We chose the OKHSL space because it is designed to provide a consistent relationship between the distance between points in the color space and the perceived distance in corresponding colors (perceptual uniformity). This is useful because it conveys the relationship between 2D point representations across all points in a relatively consistent manner. While there are many different color spaces that are designed to provide perceptual uniformity, we chose OKHSL due to its simple cylindrical shape. Many color spaces, including OKHSL, are three-dimensional and thus future work could investigate the assignment of colors from a 3D space rather than a 2D (or conical) space.

It's critical to acknowledge the constraints of the EpiVECS web-tool, particularly when it's employed for health-related analysis. Analysts must exercise caution when evaluating the outcomes from spatial data clustering methods due to potential biases introduced by spatial autocorrelation. Typically, it’s imprudent to consider time-series data from various spatial points as independent in real-world scenarios. The EpiVECS tool illustrates the cluster assignments on a choropleth map, which generally makes spatial autocorrelation evident. However, detecting more nuanced forms of autocorrelation visually can be challenging. Addressing this issue in the future could involve presenting the spatial autocorrelation of either the data or the clusters alongside the results, or by implementing clustering algorithms that account for spatial autocorrelation. A broader limitation of the EpiVECS tool is that the methods used are primarily exploratory and do not replace in-depth statistical analysis. The tool is designed to streamline the preliminary examination of complex spatiotemporal datasets, making it easier to navigate data that would be otherwise difficult to visualize at scale. There’s a rising awareness among health researchers about the importance of interactive tools that offer straightforward visualizations for intricate datasets^31,32^—EpiVECS is designed with this necessity in mind.

There has been prior interest in using cluster embedding methods to uncover patterns in spatiotemporal epidemiological data, especially for the spread of COVID-19, but those studies have been limited to self-organizing maps and haven’t provided a comparison with other methods^33,34^. An interactive tool similar to EpiVECS has been previously developed for the exploration of multivariate data over space using cluster embedding, but it did not consider methods other than self-organizing maps^35^. More broadly, several interactive visualization tools have been proposed for the exploration of data using self-organizing maps, but these typically aren’t designed for spatial analysis^36^. Together, this prior work shows the interest in using cluster embedding methods for exploration of complex data. Our work furthers the idea by considering cluster embedding methods other than self-organizing maps, as well as considering some of the challenging properties of epidemiological, spatiotemporal data. Future work could consider additional clustering techniques such as hierarchical clustering methods, which are popular in epidemiology but do not typically provide a natural high-dimensional vector representation of the clusters. That represents a challenge—and an opportunity—for a cluster embedding approach. Another direction for future work could be in the pre-processing stage: the results here show that basic time-series smoothing results in higher quality clusters; more advanced techniques could lead to further improvements. In this work, we support only a single time-series distance measure: Euclidean distance. There are many other time-series distance measures, each with unique properties^37^, and therefore future work could expand the utility of the tool by supporting more of these measures.

EpiVECS was developed as a web application in order to further its reach and reusability (in accordance with the FAIR principles^38^). The browser is a ubiquitous environment familiar to the vast majority of modern users. The requirement to install an application locally renders users less inclined to use it, in part because many users work in restricted computing environments in which they do not have permission to install software. EpiVECS is a fully in-browser application which means that the user’s data remains entirely within the sandbox of their browser and is not sent to a server. This is especially important for fields such as epidemiology where sensitive data is common. However, at present the browser is a less mature environment for unsupervised learning methods compared to other popular analytical environments such as Python and R. The web libraries often lack efficiency compared to their Python or R counterparts, both in terms of computation and memory usage. With the growing popularity of the web as a data science platform, and the maturation of new web technologies such as WebAssembly, we expect that this problem will diminish.

## Conclusion

We have investigated the use of cluster embedding methods for the exploration of spatiotemporal epidemiological data and have found that embedded k-means methods perform better than self-organizing maps on real-world data. This suggests that embedded k-means, and perhaps other embedded clustering techniques, may play an important role in future spatiotemporal data exploration. We have produced an interactive web-tool (EpiVECS) which allows users to perform cluster embedding on their own data and explore the results in an interactive dashboard. On the implementation science side, we have ensured the reusability of tool through its development as an in-browser web application.

## Methods

### Cluster embedding

A cluster embedding method partitions a dataset into clusters and provides a low-dimensional representation of each cluster. In this work, the input is a list of m-dimensional numeric vectors $X=\left[{v}_{1},{v}_{2},\dots , {v}_{n}\right]$ where ${v}_{i}\in {\mathbb{R}}^{m}$ and a desired number of clusters k. The output is a list of labels $L=[{l}_{1},{l}_{2}, \dots , {l}_{n}]$ indicating the cluster to which each vector was assigned, a list of m-dimensional representations of the clusters $C=[{c}_{1}, {c}_{2}, \dots , {c}_{k}]$ where ${c}_{i}\in {\mathbb{R}}^{m}$, and a list of two-dimensional representations of the clusters $P=[{p}_{1}, {p}_{2}, \dots , {p}_{k}]$ where ${p}_{i}\in {\mathbb{R}}^{2}$. We have compared two types of cluster embedding methods in this work: self-organizing maps and embedded k-means. In this work, we define the distance between two time-series as the Euclidean distance between their corresponding time-series vectors a and b:

$$dist\left( {a,b} \right) = \left \| a-b \right \|_2 = \sqrt {\left( {a_{1} - b_{1} } \right)^{2} + \cdots + \left( {a_{m} - b_{m} } \right)^{2} }$$

### Self-organizing maps

Self-organizing maps are an unsupervised machine learning technique typically used for dimensionality-reduction, clustering, and visualization^22,23^. They differ substantially from the artificial neural networks (ANNs) that dominate modern machine-learning. While they share the foundational concept of interconnected nodes or neurons, their architectures differ: ANNs typically have a feed-forward structure where information moves in one direction from input to output layers, whereas SOMs have interconnected nodes without distinct layers. They are also trained differently, with ANNs typically being trained using gradient descent via backpropagation whereas self-organizing maps take a competitive learning approach similar to k-means clustering. Specifically, self-organizing maps consist of a set of nodes where each node is comprised of a weight vector and a location vector. The weight vector is of equal dimensionality to the input vectors and the location vector is typically a two-dimensional vector on a grid structure. The size and shape of the grid is up to the analyst; commonly chosen shapes include rectangular and hexagonal grids. The first step in training a self-organizing map is the initialization of the weight vectors, which is typically done randomly. At a high level, training of a self-organizing map proceeds through a series of steps at each of which a vector from the input set is chosen and each of the weight vectors is moved a step closer to the chosen input vector. The input vectors are usually chosen sequentially from a random ordering of the input vectors, over several runs (epochs). The node with weight vector closest to the chosen input vector (the ‘best matching node’) takes the largest step towards the input vector, and the steps taken by the other nodes is proportional to the distances between their respective location vectors and the best matching node’s location vector. In essence, the change propagates through the grid. The size of the step taken by each vector is also dependent on the learning rate, which typically decreases across training. Training is stopped when a pre-chosen maximum number of steps is exceeded or when a convergence criterion is met. For a more detailed description of the specific self-organizing map implementation used in this work, see Data Availability. For self-organizing maps, the node weight vectors are the high-dimensional cluster representations, C and the node location vectors are the two-dimensional cluster representations P.

### Embedded k-means

An embedded k-means clustering method consists of two steps: applying k-means clustering to the input vectors, and then embedding (i.e. applying a dimensionality-reduction technique to) the centroids of the resulting clusters. The first step in k-means clustering is the initialization of the m-dimensional cluster centroids, often done randomly. Then, each input vector is assigned to the cluster whose centroid it is closest to (for this work, this means closest in Euclidean space) and the centroids are set equal to the mean of all vectors assigned to that cluster. The previous step then repeats until some stopping criterion is met—either convergence or exceeding a pre-defined maximum number of steps. The centroids are the high-dimensional cluster representations, C, introduced above. To calculate the low-dimensional cluster representations P, a dimensionality reduction algorithm is applied to the cluster centroids. In this work, we compare four dimensionality reduction methods: principal component analysis (PCA), Sammon mapping, t-SNE, and UMAP. Sammon mapping was used in the oKMC+ method which, as far as we are aware, is the first application of the embedded k-means approach to cluster embedding^26^.

### Data processing

Pre-processing the input data is an important step in ensuring it is suitable for the cluster embedding methods. A typical first step is to normalize the data. There are many different normalization methods and the choice of which to use depends on the goals of the analysis, e.g. whether the scale of the vectors are important or just their shape. A popular choice for normalizing numerical data is z-score normalization, where values are normalized so that the mean equals 0 and the standard deviation equals 1. For vector data, z-score normalization can be done row-wise, where each vector is normalized, or column-wise, where each dimension is normalized. In this work, row-wise means we are normalizing the values for each spatial location, whereas column-wise means we are normalizing the values for each time-point, and therefore we will subsequently refer to row-wise normalization as space-wise and column-wise as time-wise. We are interested in retaining the shapes of the time-series, so we have chosen to apply space-wise z-score normalization when comparing the performance of the cluster embedding methods. However, space-wise, time-wise, and combined z-score normalization methods are available as pre-processing options in the EpiVECS web-tool (see Availability).

Real-world time-series data is often noisy, and noise can have a substantial impact on the performance of clustering methods. Therefore, it can be useful to reduce noise in the data before applying a cluster embedding method. One way to reduce noise is by applying a time-series smoothing technique, the simplest of which is a moving average smoother. A moving average smoother slides a fixed-size window over a time-series and replaces the value at the center of the window with the mean of all values in the window. For a window of size 2h + 1 and vector v, the ith value in the smoothed vector $v_{i}^{\prime }$ is set as follows:

$$v_{i}^{\prime } = mean\left( {v_{i - h} , \ldots ,v_{i} , \ldots ,v_{i + h} } \right)$$

This doesn’t work at the start and end of the vector, and as is produces a smoothed vector which is shorter than the input vector. However, this can be mitigated in a number of ways, and the way we have chosen is to shorten the sliding window as needed at the ends of the vector, e.g. ${v}_{1}{\prime}=mean({v}_{1},\dots ,{v}_{i+h})$. This means that some smoothing is still performed at the edges of the vector and the initial length of the vector is maintained.

### Clustering validation metrics

#### Silhoutte score

The silhouette coefficient is a metric which quantifies the similarity of a vector to its own cluster compared to other clusters^29^. Values range from − 1 to 1, with a score of 1 indicating that a vector is well matched to its assigned cluster and poorly matched to neighboring clusters. The silhouette coefficient ${s}_{i}$ for vector ${v}_{i}$ is defined as follows:

$$s_{i} = \frac{{\left( {b_{i} - a_{i} } \right)}}{{\max \left( {a_{i} , b_{i} } \right)}}$$

where ${a}_{i}$ is the mean distance between ${v}_{i}$ and the other vectors assigned to the same cluster, and ${b}_{i}$ is the mean distance between ${v}_{i}$ and the vectors of the nearest cluster to which it was not assigned. The silhouette score ${S}_{silhoutte}$ is the mean silhouette coefficient over all vectors in the dataset.

#### Calinski-Harabasz score

The Calinski-Harabasz score is defined as the ratio of the between-cluster dispersion to the within-cluster dispersion^39^. The score takes positive values with higher values indicating better partitioned clusters:

$$S_{ch} = \frac{n - k}{{k - 1}} \cdot \frac{{\mathop \sum \nolimits_{j = 1}^{k} n_{j} \cdot \left \| c_{j} - \overline{X}\right \|^{2} }}{{\mathop \sum \nolimits_{j = 1}^{k} \mathop \sum \nolimits_{i = 1}^{{n_{j} }} \left \| G_{j,i} - c_{j} \right \|^{2} }}$$

where ${n}_{j}$ is the number of vectors in cluster j, $\overline{X }$ is the vector mean of the entire dataset, and ${G}_{j,i}$ is the i_th_ vector assigned to cluster j_._

#### Davies-Bouldin score

The Davies-Bouldin score is calculated as the average ratio of intra-cluster distance (cluster diameter) to inter-cluster distance (distance between cluster centroids)^40^. The score takes positive values, with lower values indicating better partitioned clusters:

$$S_{db} = \frac{1}{k} \cdot \mathop \sum \limits_{i = 1}^{k} \mathop {\max }\limits_{j \ne i} \frac{{r_{i} + r_{j} }}{{d_{ij} }}$$

where ${r}_{k}$ is the mean Euclidean distance between each vector in cluster k and the corresponding cluster centroid:

$$r_{k} = \frac{1}{{n_{k} }}\mathop \sum \limits_{i = 1}^{{n_{k} }} \left\| {G_{k,i} - c_{k} } \right\|_{2}$$

And ${d}_{ij}$ is the distance between cluster centroids ${c}_{i}$ and ${c}_{j}$.

#### S_Dbw index

The S_Dbw index is the sum of the mean intra-cluster scattering and the inter-cluster density^41^. The score takes positive values, with lower values indicating better partitioned clusters. The main idea is that a good clustering should exhibit a greater density of points at the center of a cluster compared to the mid-points between pairs of cluster centers. The index is defined as:

$$S\_dbw\left( k \right) = Scat\left( k \right) + Dens\_bw\left( k \right)$$

where $Scat(k)$ is the mean scattering for clusters:

$$Scat\left( k \right) = \frac{1}{k} \cdot \mathop \sum \limits_{i = 1}^{k} \frac{{\left\| {\sigma \left( {G_{i} } \right)} \right\|}}{{\left\| {\sigma \left( X \right)} \right\|}}$$

where $\sigma$ is the column-wise variance function and ${G}_{i}$ is the set of vectors assigned to cluster $i$. $Dens\_bw\left(k\right)$ is the inter-cluster density, the mean density between clusters compared to the density of the clusters:

$$Dens\_bw\left( k \right) = \frac{1}{{k \cdot \left( {k - 1} \right)}} \cdot \mathop \sum \limits_{i = 1}^{k} \left[ {\mathop \sum \limits_{j = 1,j \ne i}^{k} \frac{{\mathop \sum \nolimits_{{v \in G_{i } \cup G_{j} }} f\left( {v, u_{ij} } \right)}}{{\max \left\{ {\mathop \sum \nolimits_{{v \in G_{i} }} f\left( {v,c_{i} } \right) ,\mathop \sum \nolimits_{{v \in G_{j} }} f\left( {v,c_{j} } \right) } \right\}}}} \right]$$

where ${u}_{i,j}$ is the midpoint of ${c}_{i}$ and ${c}_{j}$, and f is a function which indicates whether the first argument vector is in the neighborhood of the second argument vector, specifically:

$$\left( {v, u} \right) = \left\{ {\begin{array}{*{20}l} {0,} \hfill & { if\, \left \| v - u \right \|_{2} > stdev} \hfill \\ {1, } \hfill & {otherwise} \hfill \\ \end{array} } \right.$$

where $stdev$ is the mean standard deviation of the clusters:

$$stdev = \frac{1}{k} \cdot \sqrt {\mathop \sum \limits_{i = 1}^{k} \left\| {\sigma \left( {G_{i} } \right)} \right\|}$$

#### Mean squared quantization error

The mean squared quantization error (MSQE) is the mean square error between each vector and the centroid of its assigned cluster:

$$MSQE = \frac{1}{n} \cdot \mathop \sum \limits_{i = 1}^{n} \left\| {v_{i} - c_{{l_{i} }} } \right\|_{2}^{2}$$

### Dimensionality-reduction validation metrics

#### Residual variance metrics

To validate the quality of the dimensionality reduction, we have used two methods based on the residual variance of the distance matrices of the original vector set and the dimensionality-reduced vector set. An element ${D}_{ij}$ in the distance matrix $D$ for the original vector set is defined as the Euclidean distance between ${v}_{i}$ and ${v}_{j}$. An element $D_{ij}^{\prime }$ in the distance matrix $D^{\prime}$ is defined similarly, but for vectors in the dimensionality-reduced vector set $P$. From these matrices we can calculate the residual variance Vr as follows:

$$Vr = r\left( {D, D^{\prime}} \right)^{2}$$

where r is the Pearson’s correlation coefficient. Vrs is calculated similarly, with r instead referring to the Spearman’s correlation coefficient.

#### Co-ranking matrix metrics

Several of the metrics used to validate the dimensionality reduction rely on the co-ranking matrix. The co-ranking matrix is derived from the ranking matrix of the cluster centroids C and the dimensionality-reduced cluster centroids P. The ranking matrix for C is a matrix R where the value ${R}_{ij}$ means that ${v}_{j}$ is the ${R}_{ij}$-th nearest neighbor of ${v}_{i}$. The ranking matrix for P is notated as ${R}_{ij}^{\prime}$ and is defined in the same manner. Each entry ${Q}_{kl}$ in the co-ranking matrix is equal to the number of vectors assigned rank $k$ in the higher-dimensionality vector set $C$ that were assigned rank $l$ in the lower dimensionality vector set $P$:

$${Q}_{kl}=\#\{\left(i,j\right):{R}_{ij}=k\; and\; {R}_{ij}^{\prime} =l\}$$

From this, trustworthiness is defined as:

$$T\left(h\right)=1-\frac{1}{n\cdot h\cdot (n-h)}\cdot \sum_{i=h}^{n}\sum_{j=1}^{h}{Q}_{ij}\cdot (i-h)$$

where h is the number of neighbors considered by the metric. Continuity is defined similarly:

$$C\left(h\right)=1-\frac{1}{n\cdot h\cdot (n-h)}\cdot \sum_{i=1}^{h}\sum_{j=h}^{n}{Q}_{ij}\cdot (j-h)$$

In the results, the AUC over values of h from 1 to $n-2$ is reported for trustworthiness and continuity.

A similar metric to trustworthiness and continuity is the co-k nearest neighbor size:

$${Q}_{NN}\left(h\right)=\frac{1}{h\cdot n}\sum_{i=1}^{h}\sum_{j=1}^{h}{Q}_{ij}$$

which is used to derive two of the metrics reported in the results, ${Q}_{local}$:

$${Q}_{local}=\frac{1}{{h}_{max} }\sum_{h=1}^{{h}_{max} }{Q}_{NN}(h)$$

and $${Q}_{global}:$$

$${Q}_{global}=\frac{1}{{n-h}_{max} }\sum_{h={h}_{max} }^{n-1 }{Q}_{NN}(h)$$

where

$${h}_{max}=argma{x}_{h} \left\{{Q}_{NN}\left(h\right)-\frac{h}{n-1}\right\}$$

### Coloring

Assigning a color to each cluster is a useful way to visualize cluster assignments, especially across multiple visualizations. When a standard clustering method is applied, there is no explicit relationship between clusters, so colors are typically assigned using a categorical color scheme. However, with cluster embedding methods the 2D representation positionally conveys relationships between the clusters, and therefore can be used to assign colors in a more informative way. In the EpiVECS tool we provide the user with two color assignment options, both based on the OKHSL color space.

The OKHSL color space is a variant on the standard HSL color space with an emphasis on perceptual uniformity^42^. Perceptual uniformity is an aspirational property of certain color spaces in which perceived differences in color are proportional to the distances between the numerical representation of those colors in the space. A color in the OKHSL space is defined by three variables: hue, saturation, and lightness. Hue and saturation are polar coordinates (angle and radius respectively) on a circular plane, lightness is the “vertical” axis of a cylinder formed by the circular planes. To color 2D points using this space, we center the color cylinder at the centroid of the points and map points onto the color space based off their angle and distance from the center. Specifically, for a set of k points P we calculate the centroid $\overline{P }$, and a radius, R, defined as the maximum Euclidean distance from the centroid across all points, $R=\mathrm{max}(\parallel {p}_{0}-\overline{P}{\parallel }_{2}, \dots ,\parallel {p}_{k}-\overline{P}{\parallel }_{2})$. For each point $p\in P$ we then calculate its vector angle to the centroid $\theta =atan2({p}_{y}-\overline{{P }_{y}} , {p}_{x}-\overline{{P }_{x}})$ and Euclidean distance to the centroid $r= \left \| p- \overline{P} \right \|_2$. These values are then normalized to the [0,1] range and passed to the OKHSL color space equation alongside a fixed lightness l:

$$color\left(p\right)=OKHSL\left(\frac{\pi +\theta }{2\pi },\frac{r}{R},l\right)$$

This produces a set of points of uniform lightness, which is useful in maintaining color space uniformity but can make it hard to distinguish between visual elements. An alternative approach is to vary the lightness with r so that points further from the centroid are lighter:

$$color\left(p\right)=OKHSL\left(\frac{\pi +\theta }{2\pi },\frac{r}{R},{l}_{l}+\frac{r}{R}\cdot ({l}_{h}-{l}_{l})\right)$$

where ${l}_{l}$ and ${l}_{h}$ are the minimum and maximum lightness respectively.

### Supplementary Information

Supplementary Information.

## Data Availability

The datasets analyzed during the current study are available in the GitHub repository, https://github.com/episphere/epivecs/. The code used to obtain this data can be found at https://observablehq.com/@siliconjazz/epvecs-data-retrieval, allowing interested readers to obtain more up-to-date versions of the data.

## Code availability

The code for the EpiVECS modules and web-tool is available at https://github.com/episphere/epivecs. The backend functionality of the tool, including the data processing, the cluster embedding, and the color assignment, are available in ES6 modules, Details on how to import and use these modules can be found in the GitHub readme. The JavaScript code used to calculate the cluster embeddings compared in the results section can be found at https://observablehq.com/@siliconjazz/epivecs-results-calculate, and the code use to calculate the clustering and dimensionality reduction metrics can be found in the ‘comparison_metrics_calculator.ipynb’ Jupyter notebook in the GitHub repository. A notebook explaining the methods with interactive elements can be found at https://observablehq.com/@siliconjazz/epivecs-cluster-embedding-on-the-web. For more details on self-organizing maps, see https://observablehq.com/@siliconjazz/self-organizing-maps. The choice to distribute much of the code through Observable Notebooks is motivated both by the power of literate programming environments for explaining concepts but also the enhanced reproducibility of reactive notebooks over traditional notebooks^43^.
