# Supplementary material for: EpiVECS: exploring spatiotemporal epidemiological data using cluster embedding and interactive visualization
Source: Sci Rep. 2023 Dec 1;13:21193. doi: 10.1038/s41598-023-48484-9 (PMC10692107; doi:10.1038/s41598-023-48484-9)
Supplement: Supplementary file 1 — Supplementary Information. [file 41598_2023_48484_MOESM1_ESM.docx]

Supplementary Information

# **Supplementary Table S1**

|  | **Time Elapsed (seconds)** | |
| --- | --- | --- |
| **Number of vectors** | **K-means+Sammon** | **Self-organizing map** |
| 1,000 | 0.11 | 0.02 |
| 10,000 | 0.47 | 0.04 |
| 100,000 | 4.21 | 0.1 |
| 1,000,000 | 42.11 | 1.00 |
| 10,000,000 | 513.40 | 11.27 |

**Performance of cluster embedding using the EpiVECS tool.** The performance of the EpiVECS cluster embedding library on artificial, randomly generated vectors with dimensionality 16, on the recommend k-means based method (K-Means+Sammon) and a self-organizing map. These readings were taken on a 2021 Apple MacBook Pro with an M1 Pro chip and 16GB of memory. The vectors are generated with uniform random values, which is not conducive to convergence and therefore the method will usually terminate faster on real-world datasets with better defined clusters. The libraries and methods used in EpiVECS operate entirely in-memory, and therefore the memory limits imposed by most modern browsers (e.g. 4GB per tab in Chrome at the time of writing) will often cause the browser to crash on datasets larger than those tested (e.g. we noted consistent crashes for a dataset of 11,000,000 vectors of dimensionality 16 when using K-Means+Sammon). This current limitation should be sufficient for most geospatial use-cases — the smallest geospatial unit defined by the US census bureau is the census block, of which there are 8,132,968 in the mainland US. However, in the future, the method implementations which are less memory-intensive would be useful to allow for larger geospatial datasets or for longer time-series. We have provided a basic tool which allows the user to stress-test the EpiVECS library on their own machine: <https://episphere.github.io/epivecs/performance_tester> .
